# First Trimester Plasma MicroRNA Levels Predict Risk of Developing Gestational Diabetes Mellitus

**DOI:** 10.3389/fendo.2022.928508

**Published:** 2022-11-11

**Authors:** Cécilia Légaré, Véronique Desgagné, Kathrine Thibeault, Frédérique White, Andrée-Anne Clément, Cédrik Poirier, Zhong Cheng Luo, Michelle S. Scott, Pierre-Étienne Jacques, Patrice Perron, Renée Guérin, Marie-France Hivert, Luigi Bouchard

**Affiliations:** ^1^ Department of Biochemistry and Functional Genomics, Faculty of Medicine and Health Sciences (FMHS), Université de Sherbrooke, Sherbrooke, QC, Canada; ^2^ Clinical Department of Laboratory Medicine, Centre intégré universitaire de santé et de services sociaux (CIUSSS) du Saguenay–Lac-St-Jean – Hôpital Universitaire de Chicoutimi, Saguenay, QC, Canada; ^3^ Département de Biologie, Faculté des Sciences, Université de Sherbrooke, Sherbrooke, QC, Canada; ^4^ Prosserman Centre for Population Health Research, Department of Obstetrics and Gynecology, Mount Sinai Hospital, Faculty of Medicine, Lunenfeld-Tanenbaum Research Institute, University of Toronto, Toronto, ON, Canada; ^5^ Centre de Recherche du Centre hospitalier universitaire de Sherbrooke (CR-CHUS), Sherbrooke, QC, Canada; ^6^ Department of Medicine, Faculty of Medicine and Health Sciences (FMHS), Université de Sherbrooke, Sherbrooke, QC, Canada; ^7^ Department of Population Medicine, Harvard Medical School, Harvard Pilgrim Health Care Institute, Boston, MA, United States; ^8^ Diabetes Unit, Massachusetts General Hospital, Boston, MA, United States

**Keywords:** biomarkers, epigenetics, next-generation sequencing, pregnancy, ribo-hormones, risk factors

## Abstract

**Aims:**

Our objective is to identify first-trimester plasmatic miRNAs associated with and predictive of GDM.

**Methods:**

We quantified miRNA using next-generation sequencing in discovery (Gen3G: n = 443/GDM = 56) and replication (3D: n = 139/GDM = 76) cohorts. We have diagnosed GDM using a 75-g oral glucose tolerance test and the IADPSG criteria. We applied stepwise logistic regression analysis among replicated miRNAs to build prediction models.

**Results:**

We identified 17 miRNAs associated with GDM development in both cohorts. The prediction performance of hsa-miR-517a-3p|hsa-miR-517b-3p, hsa-miR-218-5p, and hsa-let7a-3p was slightly better than GDM classic risk factors (age, BMI, familial history of type 2 diabetes, history of GDM or macrosomia, and HbA1c) (AUC 0.78 vs. 0.75). MiRNAs and GDM classic risk factors together further improved the prediction values [AUC 0.84 (95% CI 0.73–0.94)]. These results were replicated in 3D, although weaker predictive values were obtained. We suggest very low and higher risk GDM thresholds, which could be used to identify women who could do without a diagnostic test for GDM and women most likely to benefit from an early GDM prevention program.

**Conclusions:**

In summary, three miRNAs combined with classic GDM risk factors provide excellent prediction values, potentially strong enough to improve early detection and prevention of GDM.

## Introduction

GDM is the most common pregnancy complication with a prevalence reaching up to 25% depending on ethnicity and the diagnostic criteria (1). Both the mother and her child are affected by GDM (2, 3). Mothers are at a higher risk of preeclampsia (PE), prolonged labor, and Caesarian section (2, 3). They are also at a higher risk of recurrent GDM in future pregnancies (~48%) (4) and of developing type 2 diabetes (T2D) within 5–10 years after delivery (~40%) (5). At birth, offspring are at increased risk of shoulder dystocia, prematurity, hypoglycemia, hyperinsulinemia, hyperbilirubinemia, respiratory distress syndrome, and macrosomia (2, 3, 6). They also have a higher risk of developing obesity (7), metabolic syndrome (7), T2D (8), and hypertension (9), both as children and as adults (2, 3). Treatment of GDM is effective in preventing pregnancy complications (10), but whether it prevents long-term consequences remains unclear (7, 8, 11, 12).

GDM is usually diagnosed between the 24th and 28th week of pregnancy (13). Early identification of women at a higher risk of GDM could thus help improve follow-up and prevent long-term complications as recommended by the World Health Organization (WHO) Commission on Ending Childhood Obesity (14). Lately, circulating microRNAs (miRNAs) showed promise in identifying women with GDM. However, few studies were conducted in the first trimester (15–20), and those that were had a small number of participants, or did not offer replication in independent cohort(s). Most studies also applied a targeted approach, testing selected miRNAs limiting the possibilities of novel discovery. This limitation could be overcome using a non-targeted method such as next-generation sequencing.

miRNAs are short single-stranded RNA molecules (19–24 nucleotides) involved in post-transcriptional gene expression regulation through binding to their target messenger RNAs (mRNAs) (21). MiRNAs are stable in blood and other biologic fluids (21). Several physiological mechanisms are regulated by miRNAs including glucose homeostasis, fetal growth, and development (22). Because circulating miRNAs can regulate gene translation in distant cells, they can be considered “ribo-hormones”. MiRNAs from three clusters [chromosome 14 miRNA cluster (C14MC), chromosome 19 miRNA cluster (C19MC), and miR-371-3] are largely expressed by the trophoblasts in the placenta (22). C14MC miRNAs are more abundantly expressed at the beginning of the pregnancy, whereas their levels gradually decrease as the pregnancy progresses (22). Those expressed from the C19MC cluster on chromosome 19 progressively increase during pregnancy (22). Interestingly, placental miRNAs are secreted into maternal circulation suggesting they might contribute to fetal-maternal communication (23).

We hypothesized that the plasmatic microtranscriptomic profile in the first trimester of pregnancy is dysregulated in women who subsequently developed GDM. We also tested if some miRNA could help distinguish women at a higher risk of future GDM. Our objectives were thus to identify plasmatic miRNAs measured in the first trimester of pregnancy associated with and predictive of GDM development. We will also explore their implication in the pathophysiology of GDM with biological pathways analyses.

## Materials and methods

### Study Participants

Participants were selected from the Genetics of Glucose regulation in Gestation and Growth (Gen3G) prospective pregnancy and early life cohort, which aims to improve our comprehension of the mechanisms implicated in glucose regulation during pregnancy and fetal growth (24). Included women were ≥18 years old, with a singleton pregnancy and not taking medication affecting glucose tolerance, whereas women with diabetes (reported or detected by glucose or A1c screening) at their first trimester visit (between the 4th to 16th week of pregnancy) were excluded. A total of 854 women were followed until delivery and had a complete 75-g oral glucose tolerance test (OGTT) between the 24th and 29th week of pregnancy. International Association of Diabetes and Pregnancy Study Groups (IADPSG) guidelines (13) were applied in GDM diagnosis. For this study, we selected the 444 women of European descent (only a very small number of women were of non-European descent in Gen3G and were as thus too small to consider including them in this discovery step analysis) for which a plasma sample (500 μl) collected at the first trimester of pregnancy, and with follow-up visits at 3 or 5 years postpartum, was available. A total of 56 women developed GDM. The ethics review board of *CIUSSS de l’Estrie-CHUS* approved the study and all participants provided informed written consent.

Participants in the replication cohort were selected from the 3D cohort which was described previously (25). We selected women of European descent (women of non-European descent were excluded to ensure that this replication step is valid when compared to the discovery step using Gen3G) with plasma samples collected at the first trimester of pregnancy and complete OGTT data performed between the 24th and 28th week of pregnancy. Women with a diagnosis of pre-gestational diabetes (either Type 1 or 2) or chronic hypertension without further PE diagnosis at the first trimester of pregnancy were excluded. A total of 148 eligible women, 76 with GDM, were sampled and included in this analysis. In the 3D cohort, women were recruited at multiple university hospital centers where different GDM diagnosis procedures and criteria were applied. For the purpose of this study and to improve harmonization with Gen3G, we applied the IADPSG criteria retrospectively to categorize GDM status (including only women with complete OGTT data for both GDM and non-GDM categories).

### RNA Extraction and Library Preparation

RNA extraction and library preparation were described in the work of Légaré et al. (26). Briefly, we used the standard protocol of the MirVana PARIS kit (Thermo Fisher Scientific, catalog # AM1556) for total RNA extraction from 500 µl of plasma stored at −80°C until processing. Plasma samples were collected between the 4th and 16th week of pregnancy and randomly ordered before extraction. Total RNA was eluted in 75 μl of nuclease-free water and then precipitated with ammonium acetate and ethanol and resuspended in 5 µl of RNAse-free water as described by Burgos et al. (27). RNA samples were then randomized again before library preparation. We applied the standard protocol of the TruSeq Small RNA Sample Prep kit (Illumina, BC, Canada; catalog # RS-200-0012) adapted by Burgos et al. (27). Only half of the recommended reagents volumes for ligation (3′ and 5′ ends) of RNA samples (5 µl), reverse transcription, barcoding (index 1–48: one index per sample), and PCR amplification (15 cycles) were used to ensure the optimal ratio between reagents and RNA amount. The libraries were re-suspended in 25 μl of 10 mM tris-HCl (pH 8.5) buffer.

### Library Quality Control and Sequencing

As reported previously (26), for Gen3G miRNAs sequencing, the McGill University and Génome Québec Innovation Centre (Québec, Canada) performed the library quality control, quantification, pooling, and sequencing of the replication samples as well. Quality control of the libraries (verification of concentration, library length, and the absence of primer dimers) was done with either the Agilent High Sensitivity DNA Kit (Agilent, Mississauga, ON, Canada; catalog #5067-4626) on the Agilent 2100 Bioanalyzer or the Kapa Illumina GA with Revised Primers-SYBR Fast Universal kit (Kapa Biosystems; concentration) and the LabChip GX instrument (PerkinElmer, catalog# CLS760672; library length and absence of primer dimers). Quantitative PCR (qPCR) was used for library quantification.

For Gen3G samples, the libraries were equimolarly pooled (HiSeq 2500: 7 pM final molarity; 12 libraries with different indexes per lane; HiSeq 4000: 10 pM final molarity; 20 libraries with different indexes per lane), denatured, and clustered on single-read Illumina flow cells (catalog #GD-401-3001 and catalog #GD-410-1001) following the manufacturer’s protocol. Either an Illumina HiSeq 2500 or HiSeq 4000 sequencing platform with 50 cycles, and seven cycles indexing read, was used for sequencing. Twelve samples were extracted twice and sequenced on the two platforms. MiRNA levels were highly correlated (i.e., Pearson’s correlation coefficient ≥0.94) (26) confirming that the sequencing results obtained on both platforms do not vary significantly. For the replication cohort, the Illumina NovaSeq platform was used. Each library pool (48 libraries per lane) was loaded at 225 pM on an Illumina NovaSeq S1 lane using the Xp protocol as recommended by the manufacturer. The run was performed for 1 × 100 cycles (single-end mode).

### Bioinformatics Analysis of the Sequencing Data

The extracellular RNA processing toolkit (exceRpt) pipeline version 4.6 from Rozowsky et al. was used in the analysis of our sequencing data (28). Briefly, exceRpt removes the sequences of the adapters and bad quality reads (Phred score <20 for 80% or more of the read) with FASTX-Toolkit (28). Remaining sequences were mapped to the human genome (GRCh37) and miRbase version 21 using STAR (28). Although exceRpt was optimized for low concentration small RNA analysis, we decided not to exclude the reads mapping to the ribosomal RNAs (rRNAs) to reduce computation time because there was very little contamination by rRNA sequences in our samples (average of 1.04 ± 0.90% of total reads mapped) (26). Visualization of raw read counts was used to identify and exclude eight outlier samples in the Gen3G (seven with <500,000 and one with >25 million miRNAs reads), and nine samples with <1 million miRNAs reads in the 3D.

### Statistical Analysis

Participants’ characteristics were compared with the Kruskal–Wallis test and a Dunn’s *post-hoc* test with Bonferroni correction or Mann–Whitney U-test if only two groups were compared. The DESeq2 R package (29) was used to identify miRNAs associated with GDM. Default parameters were applied, including Wald test to assess differential expression as well as the collapseReplicates function to combine read counts from samples that were sequenced twice (n = 12). The associations between miRNAs and GDM were adjusted for the sequencing run and lane as well as gestational age at the time of plasma collection and a nominal p-value <0.05 was considered significant. Results were considered replicated in the 3D when the fold changes were in the same direction with nominal one-sided p-values <0.05. The EnhancedVolcano package was used to produce volcano plot (30).

### Prediction Modeling Analyses

Gen3G samples were randomly assigned to training (70%) and test (30%) sets. We used data from the 3D as an external replication cohort. Since about twice more read counts were obtained in the 3D as compared to the Gen3G cohort, normalized DESeq2 counts were z-score–transformed to allow applying the same GDM prediction equation to both cohorts. This strategy improves the robustness of the replication. Stepwise logistic regression analyses using only replicated miRNAs were applied on the training set to select the miRNAs independently associated with GDM. To assess whether miRNAs improve GDM prediction value over that of the GDM classic risk factors, logistic regression analyses were also conducted with classical risk factors for GDM (maternal age, BMI, familial history of T2D, history of GDM, or macrosomia) and biomarkers [HbA1c, 1-h post–50-g glucose challenge test (GCT) glucose levels] with or without miRNA levels. The pROC package (31) was applied to build receiver operating characteristic (ROC) curves on either the remaining of the Gen3G cohort (30% test set) or the 3D cohort, to assess specificity and sensitivity of the test and to compare ROC curves using the DeLong test. All statistical analyses were performed in R version 4.0.2 in RStudio-server version 1.2.1335.

### KEGG Pathway Analysis

The mirPath v.3 software (32) was used for a Kyoto Encyclopedia of Genes and Genomes (KEGG) pathway analysis of the miRNAs associated with GDM in both cohorts. Analyses were restricted to experimentally validated miRNA:mRNA interactions (Tarbase v7.0 database) (33). The default settings of mirPath v.3 were applied including a p-value threshold of 0.05, application of a false discovery rate (FDR) correction and the Fisher’s exact test (hypergeometric distribution) for enrichment analysis. The pathway union parameters were employed to merge results. 

## Results

### Participants’ Characteristics

Characteristics of study participants from both the discovery cohort (Gen3G) and the replication cohort (3D) are shown in **Table 1**. Women from the 3D were ~2 years older and had their first visit about 2 weeks later on average in their pregnancy compared to women from the Gen3G [mean: 11.9 (controls) and 11.9 (GDM) for 3D vs 9.7 (controls) and 9.5 (GDM) for Gen3G]. Women with and without GDM from both cohorts had similar BMI.

**Table 1:** Characteristics of study participants from the Gen3G and 3D (replication) cohorts.

Characteristics	Controls Gen3G (380)	GDM Gen3G (56)	Controls 3D (63)	GDM 3D (76)	Difference between groups^a^
	Mean ± SD (Range)	Mean ± SD (Range)	Mean ± SD (Range)	Mean ± SD (Range)	
First trimester variables					
Age (years)	28.32 ± 4.0 (18–41)	29.71 ± 5.6 (21–47)	31.37 ± 4.5 (23–45)	31.76 ± 4.6 (20–42)	b, c
Body mass index (kg/m^2^)	25.63 ± 5.7 (16.10–54.10)	28.16 ± 7.3 (17.80–47.10)	25.03 ± 5.3 (18.14–45.49)	27.40 ± 7.1 (16.77–47.22)	NS
Gestational age (weeks)	9.65 ± 2.2 (4.10-16.30)	9.51 ± 2.8 (5.10–15.10)	11.90 ± 1.8 (5.57–14.86)	11.94 ± 1.3 (8.43–15.57)	b, c
HbA1c (%)^d^	5.20 ± 0.3 (2.9–6.1)	5.37 ± 0.3 (4.7–5.9)	NA	NA	f
HbA1c (mmol/mol)	33.33 ± 3.1 (8–43)	35.22 ± 3.1 (28–41)	NA	NA	f
1-h post-GCT glycemia (mmol/L) ^d^	5.51 ± 1.4 (2.6–10.0)	6.70 ± 1.3 (4.5–10.2)	NA	NA	f
Second trimester variables					
Gestational age (weeks)	26.41 ± 1.0 (24.10–29.40)	26.32 ± 1.0 (24.30–28.20)	27.35 ± 1.3 (24.86–30.00)	27.12 ± 1.5 (24.43–29.71)	b, c
Fasting OGTT glycemia (mmol/L)	4.17 ± 0.3 (3.4–5.0)	4.66 ± 0.6 (3.7–7.3)	4.47 ± 0.3 (3.7–5.0)	5.02 ± 0.6 (3.8–6.6)	b, c, e, f,
1-h post-OGTT glycemia (mmol/L)	6.90 ± 1.4 (3.6–9.9)	9.85 ± 1.4 (6.3–13.0)	8.05 ± 1.3 (4.7–9.9)	10.04 ± 1.2 (7.6–12.3)	b, e, f
2-h post-OGTT glycemia (mmol/L)	5.61 ± 1.1 (3.0–8.3)	8.24 ± 1.3 (4.9–11.4)	6.43 ± 1.1 (3.8–8.4)	8.27 ± 1.3 (5.0–12.0)	b, e, f

a Kruskal–Wallis test and a Dunn’s post-hoc test with Bonferroni correction (results were considered significant at Bonferroni adjusted p-value < 0.025 or Mann–Whitney U-test if only two groups were compared).

b Controls from 3D vs. controls from Gen3G.

c GDM from 3D vs. GDM from Gen3G.

d Data available only for 51 GDM and 352 controls.

e Controls from 3D vs. GDM from 3D.

f Controls from Gen3G vs. GDM from Gen3G.

GCT, glucose challenge test; GDM, Gestational diabetes mellitus; NA, Not applicable; NS, not significant; OGTT, 75-g oral glucose tolerance test; SD, standard deviation.

### Identification of the miRNAs Associated With GDM

**Figure 1:**
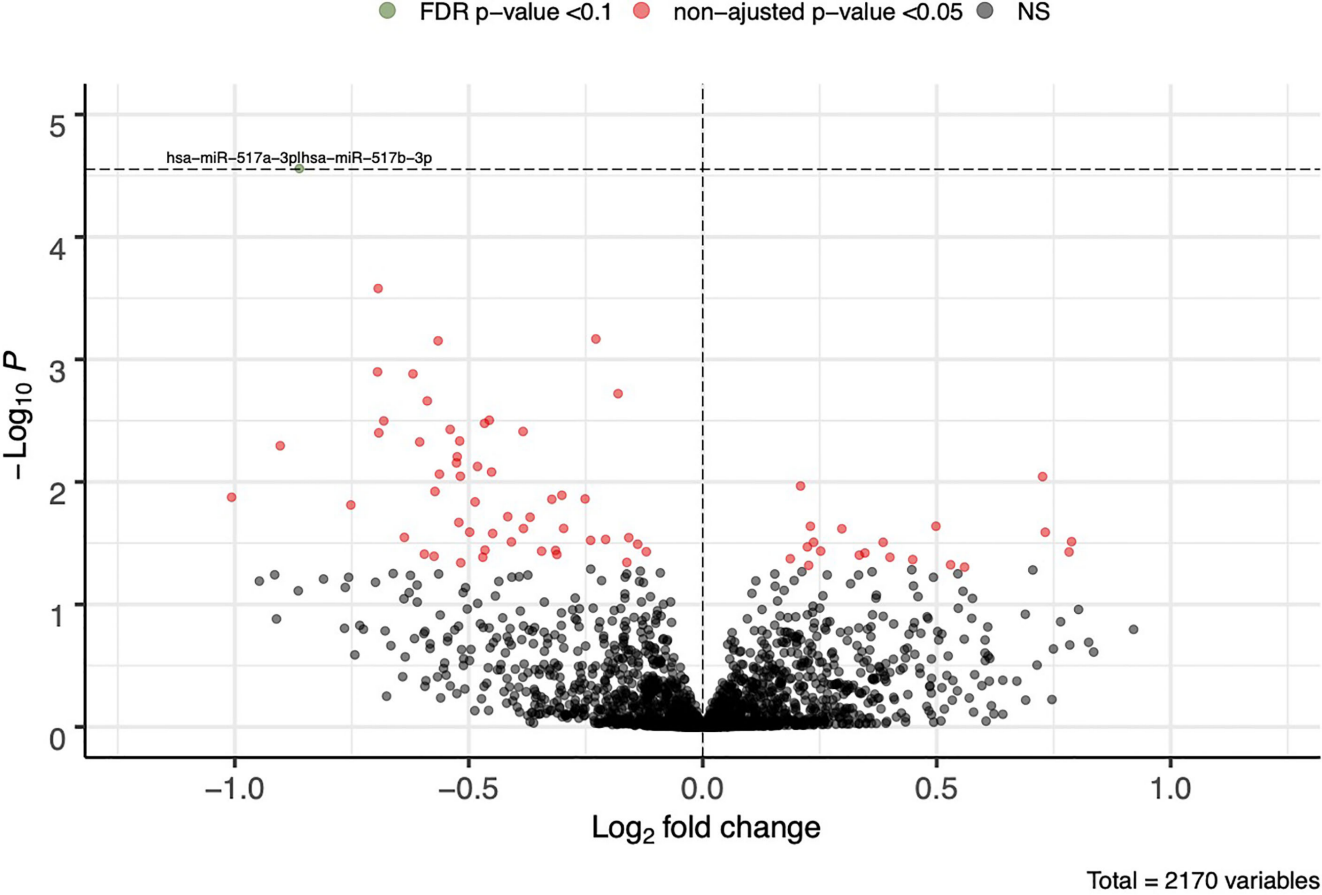
First trimester plasmatic miRNAs associated with GDM. Fold change represents the change in plasmatic miRNA abundance in GDM compared to normoglycemic women. Model adjusted for sequencing runs and lanes as well as gestational age. FDR cutoff of 0.1 is represented by a horizontal dotted line. FDR, false discovery rate; NS, non-significant.

A total of 2,170 plasmatic miRNAs were identified in Gen3G (discovery cohort; mean = 2,484 reads ± 71,800). These results were previously reported in detail (26). In Gen3G, we found 73 miRNAs nominally associated with GDM (p < 0.05). Of these, 20 were upregulated [log_2_ fold changes (L2FC) between 0.187 and 0.788] in women with GDM compared to normoglycemic women, whereas 53 were downregulated (L2FC between −1.01 and −0.121) (**Figure 1**). **Supplementary Table 1** shows the complete list of miRNAs, their mean of normalized read counts, and the percentage of women in which they were detected both in the GDM and normoglycemic groups as well as their fold change, nominal p-value and FDR q-value. One miRNA called hsa-miR-517a-3p|hsa-miR-517b-3p passed our FDR threshold (q-value < 0.1). This miRNA was 1.8 times (L2FC= −0.862) less abundant in women with GDM as compared to normoglycemic women. The findings for hsa-miR-517a-3p|hsa-miR-517b-3p were replicated in the 3D cohort (L2FC = −0.409 p = 0.023). A total of 16 additional miRNAs (23% of the total miRNAs were associated with GDM in the Gen3G) had a fold change in the same direction (lower values in GDM groups) and nominal p-value <0.05 in our replication cohort 3D (**Table 2**). The concordance in direction and nominal p-value threshold was the criteria for inclusion in the list of miRNAs considered for GDM prediction modeling (next step).

**Table 2:** First trimester plasmatic miRNAs associated with GDM in both Gen3G and 3D cohorts.

miRNAs	Gen3G controls		Gen3G GDM		Gen3G		3D controls		3D GDM		3D	
	% women	Mean ± SD	% women	Mean ± SD	L2FC	P-value	% women	Mean ± SD	% women	Mean ± SD	L2FC	P-value
**hsa-miR-517a-3p| hsa-miR-517b-3p^a^**	96.58	21.33 ± 26.07	87.50	12.41 ± 12.51	−0.862	2.76E-05	96.83	60.39 ± 46.20	98.68	41.40 ± 38.45	−0.409	0.023
**hsa-miR-141-3p**	100.00	154.42 ± 255.27	100.00	98.18 ± 62.21	−0.566	0.0007	100.00	477.57 ± 326.66	100.00	379.41 ± 416.74	−0.346	0.029
**hsa-miR-519c-3p^a^**	92.37	11.82 ± 13.41	71.43	6.04 ± 6.10	−0.695	0.0013	95.24	33.27 ± 29.49	86.84	24.75 ± 29.52	−0.474	0.036
**hsa-miR-520a-3p^a^**	99.47	91.18 ± 107.60	98.21	57.66 ± 55.98	−0.619	0.0013	98.41	180.89 ± 158.19	100.00	144.28 ± 146.03	−0.395	0.027
**hsa-miR-1323^a^**	100.00	149.59 ± 167.60	98.21	124.97 ± 104.80	−0.589	0.0022	100.00	610.56 ± 445.66	100.00	435.49 ± 390.55	−0.478	0.005
**hsa-miR-524-5p^a^**	88.16	9.12 ± 10.32	73.21	5.64 ± 5.92	−0.682	0.0032	96.83	47.58 ± 46.95	94.74	31.65 ± 40.53	−0.555	0.005
**hsa-miR-516b-5p^a^**	99.74	105.22 ± 101.37	96.43	77.36 ± 60.26	−0.540	0.0037	100.00	293.70 ± 207.06	100.00	228.52 ± 185.46	−0.343	0.029
**hsa-miR-218-5p**	76.32	6.34 ± 14.87	51.79	2.07 ± 3.84	−0.903	0.0051	90.48	11.60 ± 16.16	69.74	8.75 ± 15.29	−0.719	0.011
**hsa-miR-429**	97.11	14.09 ± 18.05	89.29	9.47 ± 7.21	−0.481	0.0075	95.24	29.28 ± 31.61	94.74	22.25 ± 23.97	−0.380	0.043
**hsa-miR-516a-5p^a^**	97.37	32.78 ± 35.83	91.07	21.24 ± 17.90	−0.518	0.0090	98.41	126.45 ± 108.98	98.68	92.73 ± 93.42	−0.348	0.047
**hsa-miR-196a-5p**	95.26	8.40 ± 7.07	91.07	7.16 ± 4.65	−0.369	0.0194	84.13	7.94 ± 7.29	76.32	5.99 ± 6.36	−0.415	0.047
**hsa-miR-215-5p**	100.00	578.70 ± 914.43	100.00	413.62 ± 236.44	−0.383	0.0240	100.00	1120.92 ± 880.97	100.00	857.98 ± 769.45	−0.357	0.023
**hsa-miR-515-3p^a^**	65.53	3.32 ± 4.81	50.00	1.82 ± 2.26	−0.638	0.0283	85.71	13.68 ± 15.29	71.05	10.35 ± 17.08	−0.632	0.020
**hsa-miR-424-5p**	99.74	49.62 ± 29.91	100.00	33.79 ± 20.65	−0.240	0.0300	98.41	117.75 ± 83.98	100.00	87.02 ± 63.01	−0.275	0.036
**hsa-let-7a-3p**	100.00	93.18 ± 27.58	100.00	71.68 ± 32.80	−0.139	0.0323	100.00	199.54 ± 93.99	100.00	166.08 ± 73.42	−0.181	0.037
**hsa-miR-525-5p^a^**	90.26	9.41 ± 10.98	80.36	6.98 ± 7.16	−0.465	0.0360	98.41	73.74 ± 64.36	93.42	50.03 ± 67.23	−0.445	0.022
**hsa-miR-518f-5p^a^**	66.58	2.78 ± 3.42	51.79	1.63 ± 2.12	−0.574	0.0405	90.48	20.68 ± 20.59	86.84	14.37 ± 15.43	−0.500	0.018

^a^C19MC miRNAs. Fold change represents difference in miRNA abundance in GDM compared to controls. % women, percentage of women for which the miRNA was detected (at least one normalized read count); C19MC, Chromosome 19 miRNA cluster; GDM, Gestational diabetes mellitus; L2FC, log2 fold changes; p-value, nominal p-value; Mean ± SD, mean and standard deviation of DESeq2 normalized reads counts.

### Plasma miRNAs Improve GDM Prediction

**Figure 2:**
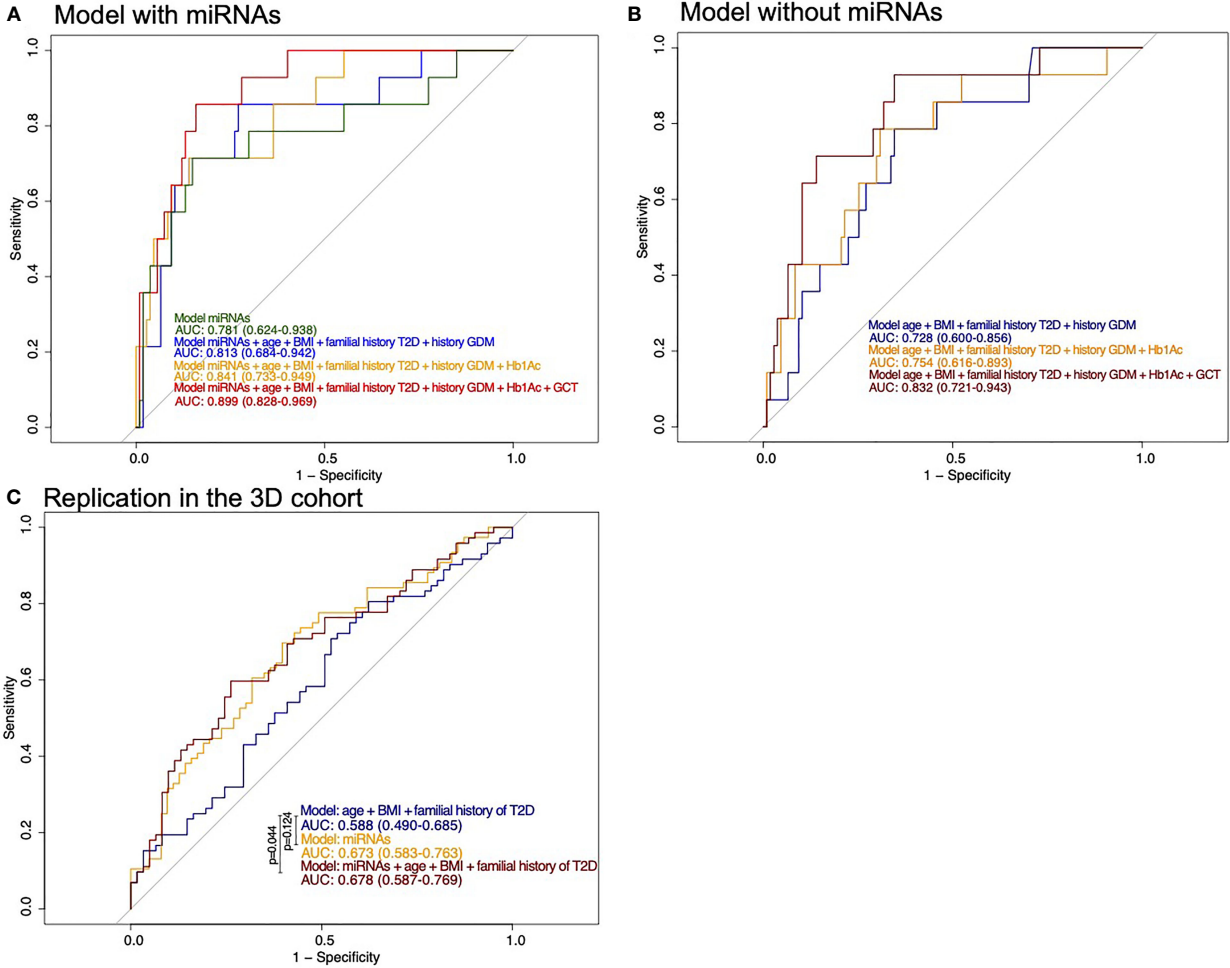
ROC curves for prediction of the risk of developing GDM. **(A)** Models including miRNAs as well as classical risk factors and biomarkers of GDM in the Gen3G cohort test set. History of GDM also includes history of macrosomia. **(B)** Models with classical risk factors and biomarkers of GDM in the Gen3G cohort test set. History of GDM also includes history of macrosomia **(C)** Models including miRNAs as well as classical risk factors of GDM in the replication cohort (3D). They are compared with the DeLong test. BMI, body mass index; GCT, 1-h post–50-g glucose challenge test value; GDM, Gestational diabetes mellitus; HbA1c, Glycated hemoglobin; T2D, Type 2 diabetes.

We first performed a stepwise analysis on the Gen3G training set using the 17 miRNAs associated with GDM in both cohorts. Three miRNAs were retained in the model: hsa-miR-517a-3p|hsa.miR-517b-3p, hsa-miR-218-5p, and hsa-let-7a-3p (**Table 3**). When tested in the Gen3G test set, these three miRNAs combined provided good discrimination with an area under the curve (AUC) of 0.78 [95% confidence interval (CI) 0.62–0.94; **Figure 2A**]. This discrimination level was similar to any combination of classic GDM risk factors without miRNAs in the Gen3G (**Figure 2B**). By sequentially adding the classic GDM risk factors and available biomarkers to the miRNA model, the overall prediction model reached up to an AUC of 0.90 (95% CI 0.83–0.97; **Figure 2A**). In addition to the three miRNAs, this model included maternal age, BMI, family history of T2D, history of GDM or macrosomia, HbA1c, and glucose levels 1-h post–50-g GCT at the first trimester. A model excluding glucose levels from the GCT (rarely used in clinical practice at the first trimester) provided an AUC of 0.84 (95% CI 0.73–0.94).

**Table 3:** Stepwise logistic regression model of miRNAs predicting GDM in the Gen3G training set.

Coefficients	Estimate	Std.Error	z value	Pr(>|z|)
**Intercept**	-2.8602	0.3609	-7.926	2.27e-15
**hsa-miR-517a-3p-has-miR-517b-3p^a^**	-0.7861	0.4387	-1.792	0.0732
**hsa-miR-218-5p^a^**	-2.4198	1.1005	-2.199	0.0279
**hsa-let-7a-3p^a^**	-0.5261	0.2067	-2.546	0.0109

**^a^** z-score of DESeq2 normalized reads counts.

**Figure 3:**
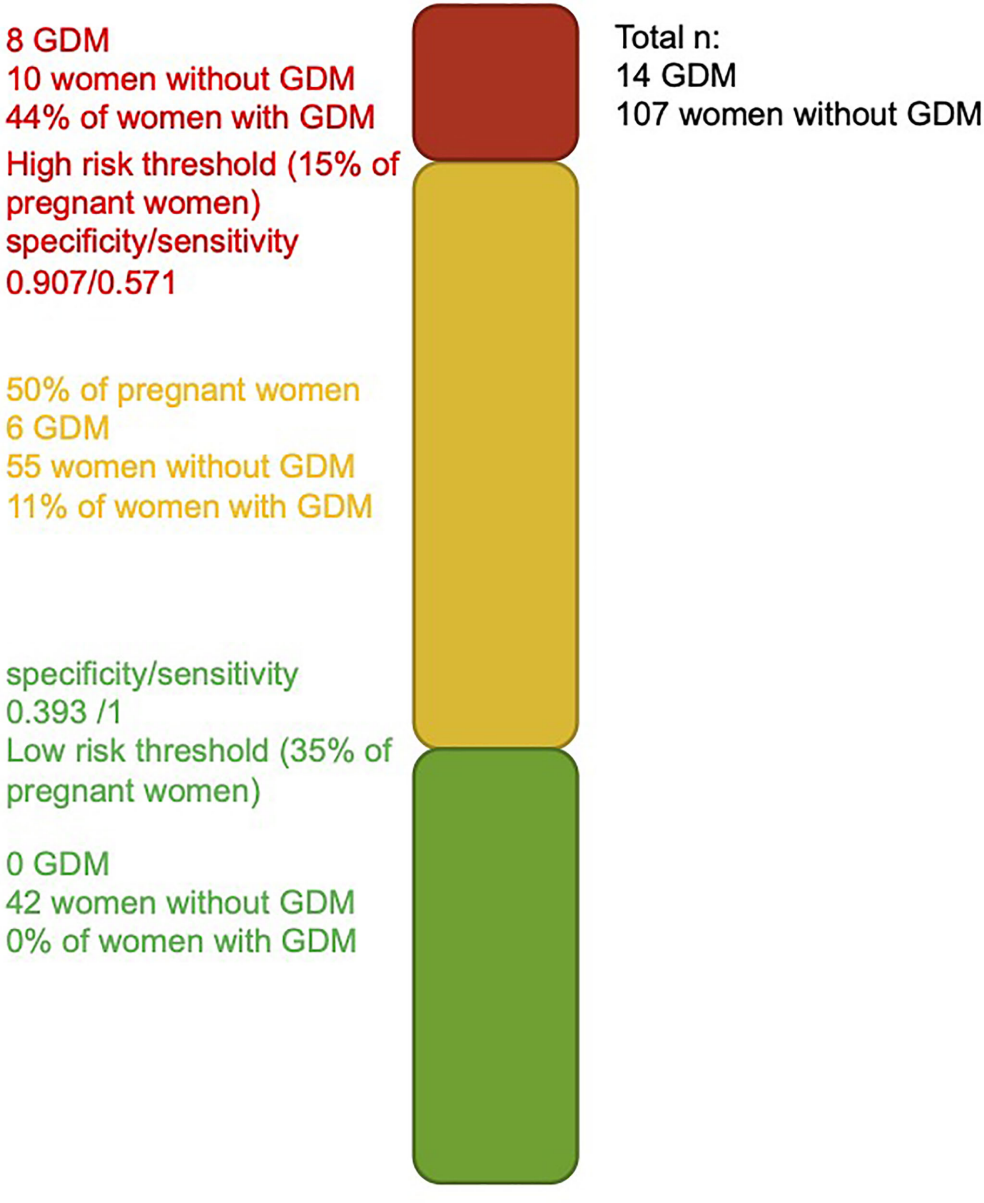
Threshold for identification of women at higher and lower risk of developing GDM. Green and red bars represent the women at a very low and higher risk of developing GDM.

Models considering miRNAs alone or their addition to GDM classical risk factors that provide higher specificity than models with classical risk factors as shown by their ROC curves are shifted to the left. The model including miRNAs, maternal age, BMI, family history of T2D, history of GDM or macrosomia, and HbA1c is the one that would be easier to implement in clinical settings. Based on this model, we suggest thresholds which identify women at a very low or higher risk of GDM. For the low-risk threshold, the goal was to exclude, with a margin, all women that developed GDM, whereas the higher risk group had to include as much as possible GDM cases while retaining an acceptable sensitivity. For the low risk, we have set the threshold at 0.036 for a specificity of 0.393 and a sensitivity of 1 (35% of women had lower values, none developed GDM): the first woman who developed GDM was ranked 49 (or 40.5%) of the 121 participants. The high-risk threshold was set at 0.269 for a specificity of 0.907 and a sensitivity of 0.571 (15% of women had higher values and 44% of them had developed GDM). Combining the high- and low-risk thresholds will leave 50% of women that will need to undergo GDM screening between the 24th and 28th week of pregnancy. These thresholds and the number of women in each group are presented in **Figure 3**.

We also applied the same equation (built on the training set of the Gen3G cohort) to 3D samples as part of our planned replication step. In the 3D, the only GDM classic risk factors available were age, BMI, and family history of T2D; together, they offer a model with a modest predicting value (AUC = 0.588; CI= 0.490–0.685). Predicting models using the three selected miRNAs combined (alone or in addition to classic risk factors) in the 3D offer predicting ability greater than chance alone (AUC ~0.67; **Figure 2C**). However, the performance of the prediction models in the 3D was overall less convincing when compared to the Gen3G results (by the AUCs).

### Biological Pathways Potentially Regulated by the miRNAs Associated with GDM

**Figure 4:**
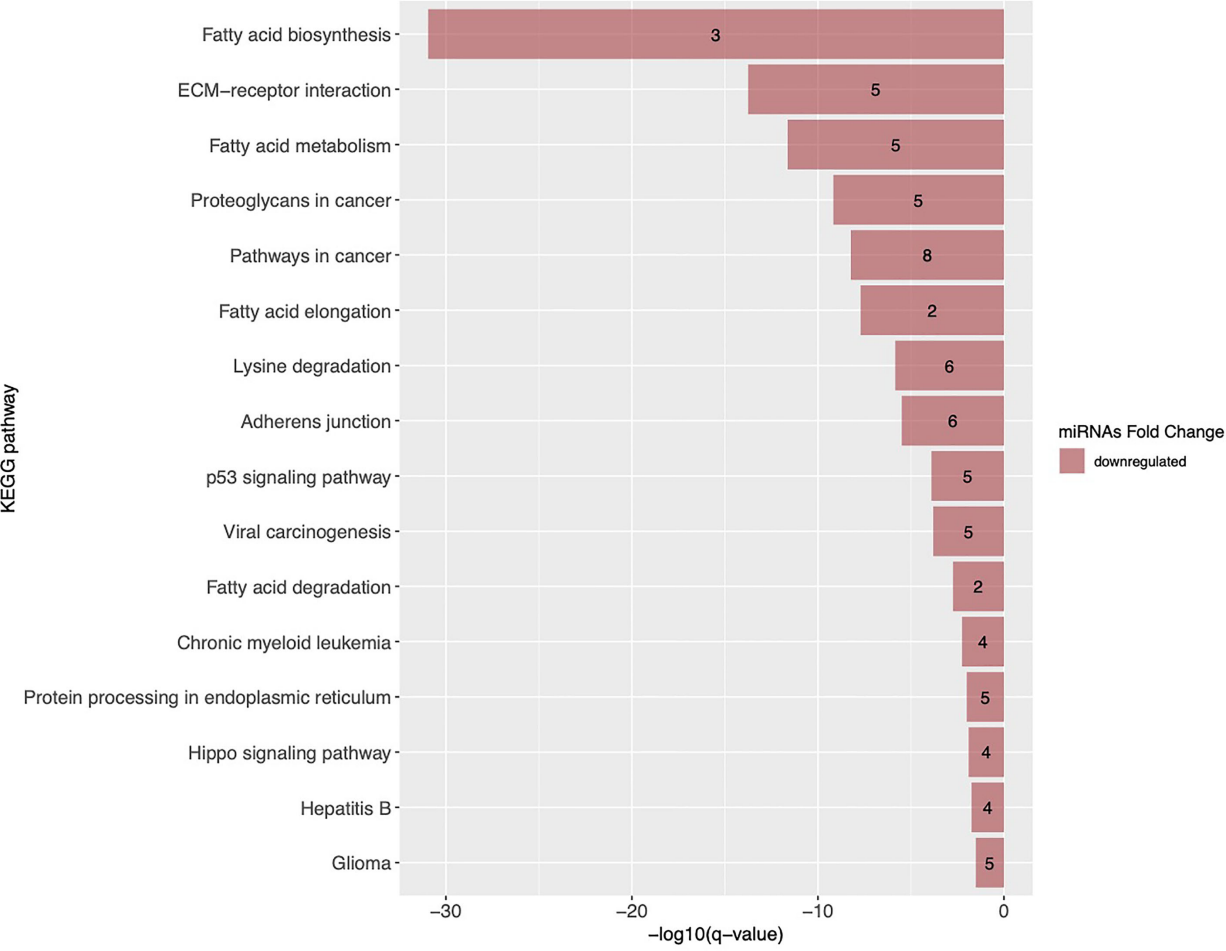
KEGG pathways targeted by miRNAs associated with GDM in both Gen3G and 3D cohorts. KEGG pathways are ranked by their FDR adjusted q-value. Pathways enriched with miRNAs negatively associated with GDM are shown as red bars and the number inside each bar represents the number of miRNAs regulating the pathway. ECM, extracellular matrix; KEGG, Kyoto Encyclopedia of Genes and Genomes.

First, we have identified 16 KEGG pathways targeted by the 17 miRNAs associated with GDM in both cohorts. This list of identified pathways is presented in **Figure 4** with the top three pathways related to lipid metabolism and extracellular matrix (ECM) receptor interaction pathways.

**Figure 5:**
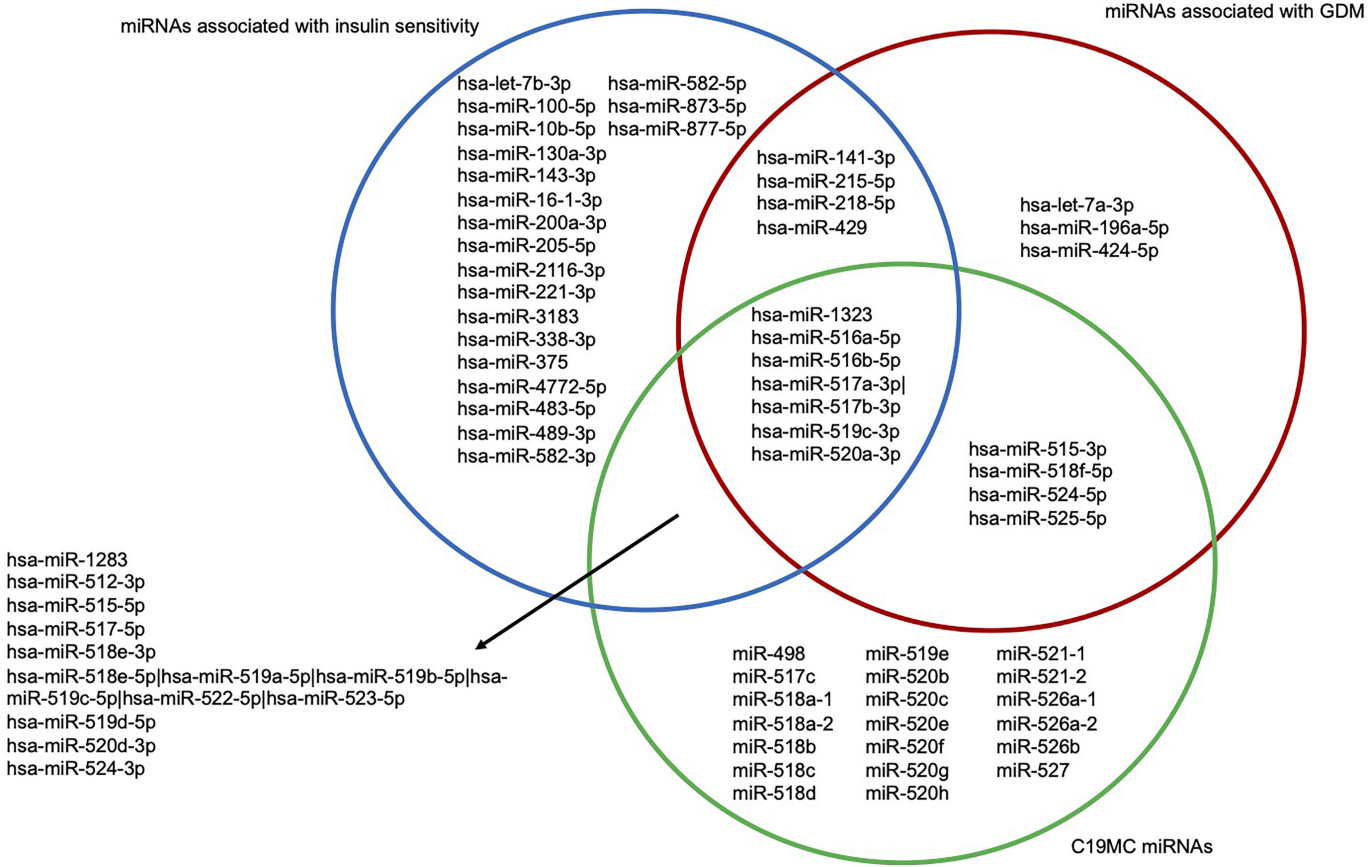
Venn diagram of first trimester miRNAs associated with GDM and insulin sensitivity as well as being expressed from the C19MC. Blue circle represents miRNAs associated with insulin sensitivity assessed with the Matsuda index between the 24th and 29th week of pregnancy. Red circle represents miRNAs associated with GDM. Green circle represents C19MC miRNAs. C19MC, Chromosome 19 miRNA cluster; GDM, Gestational diabetes mellitus.

Moreover, in another study in the Gen3G (same data set), we have recently identified miRNAs associated with insulin sensitivity estimated with the Matsuda index between the 24th and 29th week of pregnancy (34). Of the 17 miRNAs that we found associated with GDM, 10 were positively associated with insulin sensitivity: they are all decreased in GDM women in the current study (**Figure 5**).

## Discussion

In this study, we have used comprehensive and replicated microtranscriptomic data from large cohorts of plasma samples collected before the 16th week of pregnancy to identify miRNAs associated with and predictive of GDM. In brief, 17 miRNAs were associated with GDM in both cohorts, and hsa-miR-517a-3p|hsa-miR-517b-3p passed pre-specified significance threshold in the Gen3G (FDR q < 0.1) and was replicated in the 3D cohort. A prediction model with three miRNAs showed good discrimination of women at higher risk to develop GDM 3 months later, which was improved by the addition of GDM classic risk factors and two classic glycemic biomarkers.

The model based only on miRNAs performed generally well—similar to the GDM classic risk factors alone with or without HbA1c and glucose levels 1-h post-GCT. The best model (AUC ~0.90) combined miRNAs, age, BMI, familial history of T2D, history of GDM or macrosomia, HbA1c, and 1-h post–50-g GCT values. If we exclude the GCT which is rarely performed in the first trimester, the model that only included miRNAs, age, BMI, family history of T2D, history of GDM or macrosomia, and HbA1c values provides also a very strong predictive value (AUC ~0.84) and would be easier to translate into the clinical setting.

Interestingly, the comparison of the various models supports that miRNAs have more impact on the specificity of the models when compared to GDM classical risk factors alone or combination with its biomarkers. Another study used clinical measures as well as candidate biomarkers (HbA1c, random glucose, fructosamine, sex hormone binding globulin, adiponectin, and triglycerides) to predict women at risk of GDM and made several models including different variables to obtain the best prediction that could also be translated in a clinical setting (35). Contrary to our study, they only included obese women in their analyses, which is itself a risk factor for GDM. Nevertheless, their models had lower AUC (35) than ours, with their best model having an AUC of 0.77.

We have also identified thresholds that could be useful to identify women at a very low and high risk of GDM. We fully understand that the algorithm and the suggested thresholds must be validated in similar populations and replicated in populations of different ethnic origins, but, in principle, they could be used to identify women who could do without a diagnostic test for GDM and women most likely to benefit from an early GDM prevention program. The costs for the early prevention program would be partially borne by the savings made by not carrying out GDM diagnostic test in 35% of pregnant women, increasing feasibility of such program. Combining the two thresholds, only 50% of the women remain in the “gray” zone for which standard GDM screening between the 24th and 28th week of pregnancy will be needed.

Indeed, recently, there is a growing interest in using miRNAs to identify women with GDM earlier than the diagnosis that is currently used. So far, we did find nine studies (15–20, 36–38), of which six (15–20) were conducted during the first trimester of pregnancy. Three studies (15, 18, 20) also used the miRNAs they identified to predict women with GDM and two of them (15, 20) included a replication step. One study found urine hsa-miR-517a-3p|hsa-miR-517b-3p predictive of GDM but only in the second trimester of pregnancy and in the opposite direction than in the current study (19). This discrepancy could be explained by the analysis of urine instead of plasma and the timing of sampling (second vs. first trimester) as hsa-miR-517a-3p|hsa-miR-517b-3p is part of a cluster showing increasing expression from the first trimester to the end of pregnancy (19, 22). In a previous study from our group in the Gen3G, hsa-miR-517a-3p|hsa-miR-517b-3p (and hsa-miR-218-3p) measured in the first trimester was positively associated with Matsuda-insulin sensitivity index assessed between the 24th and 29th week of pregnancy (34). Hsa-miR-517a-3p|hsa-miR-517b-3p was found downregulated in women with GDM, suggesting that its expression is insufficient to exert its potential effects on insulin sensitivity and counterbalance the insulin resistance state in pregnancy. Hsa-miR-517a-3p|hsa-miR-517b-3p has been found to be downregulated in the placentas of diabetic women with fetal macrosomia (39), which is consistent with our results. Our results are in line with Santovito et al. (40) who found downregulation of let-7a in plasma of non-treated T2D patients and that its levels were increased 12 months after treatment initiation.

The exact mechanisms involved in gestational insulin resistance and GDM development in pregnancy remain poorly understood. Lipid metabolism–related pathways are of particular interest because early pregnancy is characterized by a lipogenic profile allowing to store nutrients that will be later used to meet the metabolic demand of both the mother and her growing fetus during pregnancy (41). By targeting fatty acid biosynthesis and metabolism, some pregnancy-associated miRNAs (5 miRNAs out of 17) could play a role in the regulation of the lipid metabolism pathways which might have be related to decreasing insulin sensitivity later in pregnancy.

Moreover, 10 of the 17 miRNAs that we found to be less abundant in women with GDM were also positively associated with insulin sensitivity assessed between the 24th and 29th week of pregnancy (34). In brief, our miRNAs could be implicated in the pathophysiology of GDM through their roles in lipid metabolism and insulin sensitivity regulation.

Remarkably, 10 of the 17 miRNAs associated with GDM were from the C19MC, a miRNA cluster mainly expressed in the placenta (trophoblasts) (22) that could contribute to feto-maternal communication and consequently to metabolic adaptation in pregnancy when secreted into maternal circulation (**Figure 5**) (23). All 10 miRNAs were less abundant in GDM compared to normoglycemic women.

## Strengths and limitations

To the best of our knowledge, this is the first study including the largest number of pregnant women and using miRNA sequencing as an agnostic approach. We replicated our results and prediction algorithm in a completely independent cohort to demonstrate external validity and help assess generalizability (3D first-trimester samples were collected at random, non-fasting at ~11 weeks). Gen3G samples were collected mostly following a 50-g GCT in the first trimester. The effect of a 50-g GCT on miRNA expression is not currently known, but because the results were replicated in the 3D cohort, we believe that our models are robust. Also, we did not have access to as many clinical characteristics in the 3D cohort, which could also explain why the model’s AUC values were not as high as in Gen3G. Our study has also other limitations. We did not validate our results using another method such as qPCR or digital PCR, which will be needed if clinical applications are intended. Also, replication in other ethnicities will be needed to confirm our miRNA selection and also their weight in the algorithm in other population. Our prediction algorithm performed better when other clinical values (HbA1c and 1-h post–50-g GCT) were considered, but we were not able to confirm these results in the 3D as these were not available.

## Conclusion

We have identified plasmatic miRNAs measured between the 4th and 16th week of pregnancy that are associated with subsequent development of GDM. Three of these miRNAs could be used in combination with GDM classical risk factors to identify women at a low or higher risk of GDM 12 weeks on average prior to its current diagnostic. This would allow to decrease the need for GDM screening between 24th and 28th week for women at low risk and opens the window for early GDM prevention.

## Data Availability Statement

The original contributions presented in the study are publicly available. This data can be found here: GEO repository, accession numbers GSE216275 and GSE216997. Further inquiries can be directed to the corresponding author.

## Ethics Statement

The studies involving human participants were reviewed and approved by Centre intégré universitaire de santé et de services sociaux de l’Estrie-CHUS, Sherbrooke, Canada. The patients/participants provided their written informed consent to participate in this study.

## Author Contributions

CL was responsible for data collection, statistical analysis, writing of the manuscript and contributes to bioinformatics analysis. VD, CP, and KT contributed to data collection and manuscript revision. FW and A-AC contributed to bioinformatics analyses and the revision of the manuscript. MS, ZL, and P-EJ contributed to data analyses and manuscript revision. PP, M-FH, RG, and LB elaborated the study design, supervised all steps of the study, and participated in manuscript writing and revision. LB is the guarantor of this work. All authors contributed to the article and approved the submitted version.

## Funding

CL, KT and A-AC are supported by doctoral research award from *Fonds de la recherche du Québec en santé* (FRQS). VD received funding from *Diabète Québec*. MS, P-EJ and LB are research scholar from the FRQS and members of the CR-CHUS, a FRQS-funded Research Center. This study was supported by the Canadian Institutes of Health Research (Grant #IGH-155183).

## Conflict of Interest

The authors declare that the research was conducted in the absence of any commercial or financial relationships that could be construed as a potential conflict of interest.

## Publisher’s Note

All claims expressed in this article are solely those of the authors and do not necessarily represent those of their affiliated organizations, or those of the publisher, the editors and the reviewers. Any product that may be evaluated in this article, or claim that may be made by its manufacturer, is not guaranteed or endorsed by the publisher.

## Abbreviations

BMI, Body mass index; C14MC, Chromosome 14 microRNA cluster; C19MC, Chromosome 19 microRNA cluster; CI, Confidence interval; ECM, extracellular matrix; L2FC, Log2 Fold change; FDR, False discovery rate; GCT, Glucose challenge test; GDM, Gestational diabetes mellitus; Gen3G, Genetics of Glucose regulation in Gestation and Growth; GH, Gestational hypertension; HbA1c, Glycated hemoglobin; IADPSG, International Association of the Diabetes and Pregnancy Study Groups; KEGG, Kyoto Encyclopedia of Genes and Genomes; miRNA, MicroRNA; mRNA, Messenger RNA; npv, negative predictive value; OGTT, Oral glucose tolerance test; PE, Preeclampsia; ppv, positive predictive value; ROC, receiver operating characteristic; rRNA, Ribosomal RNA; T2D, Type 2 diabetes; WHO, World Health Organization.
