# Supplementary material for: First Trimester Plasma MicroRNA Levels Predict Risk of Developing Gestational Diabetes Mellitus
Source: Front Endocrinol (Lausanne). 2022 Nov 11;13:928508. doi: 10.3389/fendo.2022.928508 (PMC9693764; doi:10.3389/fendo.2022.928508)
Supplement: Supplementary file 1 [file Table_1.docx]

Supplementary Table 1. The complete list of miRNAs associated with GDM in Gen3G

| **miRNAs** | **Gen3G controls** | | **Gen3G GDM** | | **Gen3G** | | |
| --- | --- | --- | --- | --- | --- | --- | --- |
|  | **% women** | **Mean ± SD** | **% women** | **Mean ± SD** | **L2FC** | **pvalue** | **FDR q-value** |
| **hsa-miR-517a-3p\|hsa-miR-517b-3p^b^** | 96.58 | 21.33 ± 26.07 | 87.50 | 12.41 ± 12.51 | -0.862 | 2.76E-05 | 0.06 |
| **hsa-miR-512-3p^b^** | 100.00 | 297.18 ± 399.81 | 100.00 | 218.15 ± 179.95 | -0.694 | 0.0003 | 0.29 |
| **hsa-miR-338-3p** | 100.00 | 112.21 ± 35.34 | 100.00 | 82.97 ± 31.00 | -0.228 | 0.0007 | 0.38 |
| **hsa-miR-141-3p** | 100.00 | 154.42 ± 255.27 | 100.00 | 98.18 ± 62.21 | -0.566 | 0.0007 | 0.38 |
| **hsa-miR-519c-3p^b^** | 92.37 | 11.82 ± 13.41 | 71.43 | 6.04 ± 6.10 | -0.695 | 0.0013 | 0.47 |
| **hsa-miR-520a-3p^b^** | 99.47 | 91.18 ± 107.60 | 98.21 | 57.66 ± 55.98 | -0.619 | 0.0013 | 0.47 |
| **hsa-miR-548av-5p\|hsa-miR-548k** | 100.00 | 148.19 ± 36.82 | 100.00 | 124.77 ± 34.01 | -0.181 | 0.0019 | 0.59 |
| **hsa-miR-1323^b^** | 100.00 | 149.59 ± 167.60 | 98.21 | 124.97 ± 104.80 | -0.589 | 0.0022 | 0.59 |
| **hsa-miR-149-5p** | 95.53 | 7.07 ± 4.69 | 83.93 | 4.45 ± 3.28 | -0.456 | 0.0031 | 0.62 |
| **hsa-miR-524-5p^b^** | 88.16 | 9.12 ± 10.32 | 73.21 | 5.64 ± 5.92 | -0.682 | 0.0032 | 0.62 |
| **hsa-miR-548j-3p** | 93.68 | 8.64 ± 5.96 | 91.07 | 5.70 ± 3.95 | -0.466 | 0.0033 | 0.62 |
| **hsa-miR-516b-5p^b^** | 99.74 | 105.22 ± 101.37 | 96.43 | 77.36 ± 60.26 | -0.540 | 0.0037 | 0.62 |
| **hsa-miR-577** | 98.95 | 16.62 ± 9.97 | 91.07 | 9.80 ± 6.68 | -0.384 | 0.0039 | 0.62 |
| **hsa-miR-518a-5p\|hsa-miR-527^b^** | 82.63 | 6.02 ± 7.36 | 67.86 | 3.48 ± 4.87 | -0.692 | 0.0040 | 0.62 |
| **hsa-miR-518e-5p\|hsa-miR-519a-5p\|hsa-miR-519b-5p\|hsa-miR-519c-5p\|hsa-miR-522-5p\|hsa-miR-523-5p^b^** | 99.21 | 45.02 ± 48.06 | 94.64 | 37.37 ± 29.96 | -0.519 | 0.0046 | 0.64 |
| **hsa-miR-517-5p^b^** | 94.74 | 17.65 ± 20.18 | 82.14 | 11.85 ± 11.46 | -0.605 | 0.0047 | 0.64 |
| **hsa-miR-218-5p** | 76.32 | 6.34 ± 14.87 | 51.79 | 2.07 ± 3.84 | -0.903 | 0.0051 | 0.65 |
| **hsa-miR-1283^b^** | 98.68 | 58.94 ± 62.31 | 96.43 | 46.06 ± 38.13 | -0.525 | 0.0062 | 0.75 |
| **hsa-miR-375** | 100.00 | 1920.58 ± 4961.74 | 100.00 | 2068.51± 1783.47 | -0.526 | 0.0070 | 0.80 |
| **hsa-miR-429** | 97.11 | 14.09 ± 18.05 | 89.29 | 9.47 ± 7.21 | -0.481 | 0.0075 | 0.81 |
| **hsa-miR-200a-3p** | 99.47 | 30.39 ± 47.42 | 100.00 | 22.29 ± 14.68 | -0.451 | 0.0083 | 0.82 |
| **hsa-miR-515-5p^b^** | 91.84 | 11.07 ± 13.34 | 75.00 | 6.64 ± 6.84 | -0.563 | 0.0086 | 0.82 |
| **hsa-miR-516a-5p^b^** | 97.37 | 32.78 ± 35.83 | 91.07 | 21.24 ± 17.90 | -0.518 | 0.0090 | 0.82 |
| **hsa-miR-4477b** | 42.37 | 1.18 ± 1.52 | 48.21 | 1.66 ± 2.22 | 0.726 | 0.0090 | 0.82 |
| **hsa-miR-423-5p** | 100.00 | 82960.11 ± 34663.05 | 100.00 | 105641.15 ± 41377.17 | 0.209 | 0.0108 | 0.94 |
| **hsa-miR-1285-5p** | 73.16 | 2.48 ± 2.37 | 53.57 | 2.04 ± 2.57 | -0.572 | 0.0120 | 1.00 |
| **hsa-miR-145-5p** | 100.00 | 98.72 ± 67.12 | 100.00 | 68.84 ± 38.01 | -0.301 | 0.0129 | 1.00 |
| **hsa-miR-154-3p^a^** | 31.84 | 0.94 ± 1.65 | 7.14 | 0.26 ± 0.65 | -1.007 | 0.0133 | 1.00 |
| **hsa-miR-1277-5p** | 99.74 | 139.23 ± 60.49 | 100.00 | 91.63 ± 51.36 | -0.251 | 0.0138 | 1.00 |
| **hsa-miR-200c-3p** | 98.42 | 11.88 ± 6.46 | 96.43 | 9.72 ± 6.06 | -0.323 | 0.0139 | 1.00 |
| **hsa-miR-4433b-3p** | 99.74 | 37.50 ± 42.83 | 100.00 | 39.37 ± 43.11 | -0.487 | 0.0146 | 1.00 |
| **hsa-miR-1256** | 51.32 | 1.58 ± 1.94 | 30.36 | 0.87 ± 1.29 | -0.752 | 0.0154 | 1.00 |
| **hsa-miR-1277-3p** | 85.53 | 4.67 ± 3.35 | 60.71 | 2.45 ± 2.56 | -0.417 | 0.0192 | 1.00 |
| **hsa-miR-196a-5p** | 95.26 | 8.40 ± 7.07 | 91.07 | 7.16 ± 4.65 | -0.369 | 0.0194 | 1.00 |
| **hsa-miR-373-3p^c^** | 94.47 | 19.84 ± 37.44 | 91.07 | 20.34 ± 27.87 | -0.521 | 0.0214 | 1.00 |
| **hsa-miR-3160-3p** | 61.84 | 1.94 ± 1.97 | 69.64 | 2.36 ± 2.53 | 0.498 | 0.0230 | 1.00 |
| **hsa-miR-501-3p** | 100.00 | 38.63 ± 23.14 | 100.00 | 69.28 ± 49.58 | 0.230 | 0.0230 | 1.00 |
| **hsa-miR-10a-3p** | 99.21 | 14.24 ± 10.97 | 94.64 | 11.16 ± 6.29 | -0.297 | 0.0240 | 1.00 |
| **hsa-miR-215-5p** | 100.00 | 578.70 ± 914.43 | 100.00 | 413.62 ± 236.44 | -0.383 | 0.0240 | 1.00 |
| **hsa-miR-610** | 96.58 | 7.75 ± 4.56 | 98.21 | 11.44 ± 8.95 | 0.297 | 0.0241 | 1.00 |
| **hsa-miR-518e-3p^b^** | 90.26 | 8.28 ± 9.57 | 78.57 | 5.98 ± 6.34 | -0.498 | 0.0257 | 1.00 |
| **hsa-miR-196b-3p** | 38.68 | 1.32 ± 2.25 | 37.50 | 1.53 ± 2.14 | 0.732 | 0.0257 | 1.00 |
| **hsa-miR-526b-5p^b^** | 95.79 | 17.42 ± 17.24 | 87.50 | 14.98 ± 15.23 | -0.449 | 0.0263 | 1.00 |
| **hsa-miR-515-3p^b^** | 65.53 | 3.32 ± 4.81 | 50.00 | 1.82 ± 2.26 | -0.638 | 0.0283 | 1.00 |
| **hsa-miR-199a-3p\|hsa-miR-199b-3p** | 100.00 | 12800.25± 4074.98 | 100.00 | 10331.57 ± 3946.85 | -0.158 | 0.0285 | 1.00 |
| **hsa-miR-143-3p** | 100.00 | 31759.36 ± 14319.23 | 100.00 | 28049.68 ± 14333.50 | -0.208 | 0.0295 | 1.00 |
| **hsa-miR-424-5p** | 99.74 | 49.62 ± 29.91 | 100.00 | 33.79 ± 20.65 | -0.240 | 0.0300 | 1.00 |
| **hsa-miR-4482-3p** | 64.74 | 7.65 ± 15.70 | 69.64 | 9.89 ± 13.50 | 0.788 | 0.0307 | 1.00 |
| **hsa-miR-664b-5p** | 88.42 | 6.77 ± 6.56 | 91.07 | 9.92 ± 10.92 | -0.409 | 0.0309 | 1.00 |
| **hsa-miR-30c-2-3p** | 99.74 | 15.34 ± 7.87 | 100.00 | 17.35 ± 7.84 | 0.237 | 0.0311 | 1.00 |
| **hsa-miR-4516** | 90.53 | 6.81 ± 7.35 | 98.21 | 11.76 ± 10.87 | 0.385 | 0.0311 | 1.00 |
| **hsa-let-7a-3p** | 100.00 | 93.18 ± 27.58 | 100.00 | 71.68 ± 32.80 | -0.139 | 0.0323 | 1.00 |
| **hsa-miR-3605-5p** | 100.00 | 30.87 ± 16.97 | 100.00 | 40.07 ± 20.44 | 0.224 | 0.0339 | 1.00 |
| **hsa-miR-525-5p^b^** | 90.26 | 9.41 ± 10.98 | 80.36 | 6.98 ± 7.16 | -0.465 | 0.0360 | 1.00 |
| **hsa-miR-548k** | 91.05 | 4.76 ± 3.05 | 89.29 | 3.83 ± 2.92 | -0.315 | 0.0362 | 1.00 |
| **hsa-miR-18a-3p** | 100.00 | 167.68 ± 99.87 | 100.00 | 230.72 ± 216.48 | 0.252 | 0.0367 | 1.00 |
| **hsa-miR-487a-3p^a^** | 99.47 | 43.07 ± 34.17 | 100.00 | 29.25 ± 19.89 | -0.344 | 0.0368 | 1.00 |
| **hsa-miR-181c-5p** | 100.00 | 1979.37 ± 521.67 | 100.00 | 1612.76 ± 448.50 | -0.121 | 0.0371 | 1.00 |
| **hsa-miR-3936** | 24.47 | 0.64 ± 1.20 | 44.64 | 1.27 ± 1.68 | 0.783 | 0.0373 | 1.00 |
| **hsa-miR-3191-3p** | 80.26 | 3.02 ± 2.42 | 83.93 | 5.03 ± 4.38 | 0.347 | 0.0380 | 1.00 |
| **hsa-miR-520a-5p^b^** | 67.37 | 3.21 ± 4.27 | 55.36 | 2.07 ± 2.29 | -0.595 | 0.0388 | 1.00 |
| **hsa-miR-153-3p** | 93.16 | 5.70 ± 3.63 | 78.57 | 4.15 ± 4.07 | -0.312 | 0.0391 | 1.00 |
| **hsa-miR-486-5p** | 100.00 | 2286049.31± 1939372.20 | 100.00 | 3495580.77 ± 3243099.86 | 0.335 | 0.0396 | 1.00 |
| **hsa-miR-518f-5p^b^** | 66.58 | 2.78 ± 3.42 | 51.79 | 1.63 ± 2.12 | -0.574 | 0.0405 | 1.00 |
| **hsa-miR-520d-5p^b^** | 83.95 | 6.24 ± 6.53 | 66.07 | 3.79 ± 3.87 | -0.470 | 0.0412 | 1.00 |
| **hsa-miR-1255b-5p** | 93.68 | 10.32 ± 10.06 | 94.64 | 17.77 ± 18.19 | 0.400 | 0.0412 | 1.00 |
| **hsa-miR-3913-5p** | 100.00 | 21.53 ± 9.52 | 100.00 | 24.51 ± 9.41 | 0.187 | 0.0424 | 1.00 |
| **hsa-let-7c** | 73.42 | 4.15 ± 5.09 | 85.71 | 7.20 ± 9.56 | 0.449 | 0.0430 | 1.00 |
| **hsa-miR-30b-5p** | 100.00 | 4814.59 ± 1818.18 | 100.00 | 3308.52 ± 1467.52 | -0.162 | 0.0454 | 1.00 |
| **hsa-miR-371a-5p^c^** | 86.05 | 11.22 ± 13.24 | 80.36 | 10.05 ± 14.21 | -0.517 | 0.0457 | 1.00 |
| **hsa-miR-6783-5p** | 45.79 | 1.30 ± 1.64 | 55.36 | 1.83 ± 2.36 | 0.530 | 0.0475 | 1.00 |
| **hsa-miR-3127-5p** | 99.74 | 13.99 ± 9.10 | 100.00 | 23.77 ± 16.27 | 0.226 | 0.0480 | 1.00 |
| **hsa-miR-3183** | 46.32 | 1.32 ± 1.65 | 51.79 | 2.27 ± 4.79 | 0.559 | 0.0496 | 1.00 |

^a^ C14MC, ^b^ C19MC, ^c^ miR-371-3 cluster. Log2FCrepresents difference in miRNA abundance in GDM compared to controls. Abbreviations: % women: percentage of women for which the miRNA was detected (at least one normalized read count), C14MC: Chromosome 14 miRNA cluster, C19MC: Chromosome 19 miRNA cluster, FDR q-value: False discovery rate adjusted q-value, GDM: gestational diabetes mellitus, L2FC: log2 fold change, p-value: nominal p-value, Mean ± SD: mean and standard deviation of DESeq2 normalized reads counts.
